# Predation scars may influence host susceptibility to pathogens: evaluating the role of corallivores as vectors of coral disease

**DOI:** 10.1038/s41598-018-23361-y

**Published:** 2018-03-27

**Authors:** K. J. Nicolet, K. M. Chong-Seng, M. S. Pratchett, B. L. Willis, M. O. Hoogenboom

**Affiliations:** 1 College of Science and Engineering, James Cook University, Townsville, QLD 4811 Australia; 2 ARC Centre of Excellence for Coral Reef Studies, James Cook University, Townsville, QLD 4811 Australia; 3 AIMS@JCU, Townsville, 4811 Australia

## Abstract

Infectious diseases not regulated by host density, such as vector-borne diseases, have the potential to drive population declines and extinctions. Here we test the vector potential of the snail *Drupella* sp. and butterflyfish *Chaetodon plebeius* for two coral diseases, black band (BBD) and brown band (BrB) disease. *Drupella* transmitted BrB to healthy corals in 40% of cases immediately following feeding on infected corals, and even in 12% of cases 12 and 24 hours following feeding. However, *Drupella* was unable to transmit BBD in either transmission treatment. In a field experiment testing the vector potential of naturally-occurring fish assemblages, equivalent numbers of caged and uncaged coral fragments became infected with either BrB, BBD or skeletal eroding band, indicating that corallivorous fish were unlikely to have caused transmission. In aquaria, *C*. *plebeius* did not transmit either BBD or BrB, even following extended feeding on both infected and healthy nubbins. A literature review confirmed only four known coral disease vectors, all invertebrates, corroborating our conclusion that polyp-feeding fishes are unlikely to be vectors of coral diseases. This potentially because polyp-feeding fishes produce shallow lesions, not allowing pathogens to invade coral tissues. In contrast, corallivorous invertebrates that create deeper feeding scars increase pathogens transmission.

## Introduction

Infectious diseases, defined as health disorders caused by pathogenic biological agents, affect all living organisms, with detrimental consequences for host species, ecosystem function and biodiversity^1–3^. Until the late 1970s, it was generally thought that “well-adapted” parasites would cause negligible harm to their hosts^4^. Modelling studies, for example, suggested that pathogens would be lost before host populations went extinct, because pathogens would drive their hosts below a density threshold critical for disease persistence^5^. Consequently, the role of infectious disease as a driver of host population dynamics has been underappreciated, and diseases have rarely been considered to contribute significantly to animal extinctions^4^. When a disease is density-dependent, transmission increases as population density increases because of the increased probability of contact between infected and susceptible individuals. In airborne diseases (e.g., viral influenza), the likelihood of an individual becoming infected depends on the number of individuals per unit area (i.e., population density). However, some diseases are transmitted as a function of the proportion of infected *versus* uninfected individuals in the population (‘frequency-dependent’) regardless of the density of individuals^6^. When a disease is frequency-dependent, transmission increases as the proportion of infected individuals increases regardless of host density. Vector-borne pathogens and sexually-transmitted diseases are commonly frequency-dependent, and their prevalence can continue to increase even when host density is low, leading to disease-mediated population declines and extinctions^7,8^. The same is true when pathogens remain viable outside of their hosts, in a ‘reservoir’, or when pathogens are able to infect multiple hosts, both of which release pathogens from the dynamics of a specific one host-one pathogen system^9,10^.

The potential of corallivores to act as vectors for diseases that infect reef-building (scleractinian) corals is a cause for concern given drastic declines in coral populations over the past 50 years and the functional loss of up to 25% of coral reefs globally^11^. Extensive coral loss and degradation of reef ecosystems is largely attributed to overfishing, pollution, coastal development and climate change^12^, with only limited losses attributed to infectious diseases. However, there is increasing evidence that coral diseases are an important contributor to the global degradation of coral reef ecosystems. Maynard *et al*.^13^ predicts that “increases in the prevalence and severity of coral diseases will be a major future driver of decline and changes in coral reef community composition”, given projections of how rising sea temperatures are likely to affect pathogen virulence and host susceptibility. Such projections are consistent with evidence that changes in host-pathogen interactions following environmental and/or ecosystem modification have been key to the emergence of most infectious diseases^14^. For instance, the devastating sea star wasting disease is thought to have emerged due to warming sea temperatures^15^.

Most coral diseases, in both the Caribbean^16^ and the Indo-Pacific^17^, affect multiple coral species. For example, black band disease (BBD) affects at least 40 coral species on the Great Barrier Reef^17,18^, enabling the disease to circumvent more typical density-dependent, host-pathogen dynamics. Moreover, some coral pathogens have reservoirs and vectors that maintain pathogen loads, even when host population densities are low. The coral disease white pox, for example, is caused by the pathogen *Serratia marescens*, which survives and remains virulent within the corallivorous snail *Coralliophila abbreviata*^19^, enabling the snail to infect new coral colonies. Studies of disease vectors can be undertaken even before a pathogen has been formally identified and are critical in cases where vaccination and quarantine programs are difficult or impossible, such as for coral populations. Malaria, for example, is a well-known disease that is managed primarily by vector control via insecticide spraying and/or mosquito habitat reduction^20^. Ultimately, a good understanding of a vector’s identity, and the timeframes and biological processes involved in the transmission process, are required to establish control procedures in disease management for syndromes with known or unknown pathogens.

Black band disease (BBD) and brown band disease (BrB) are among the most conspicuous and widespread coral diseases found on Australia’s Great Barrier Reef (GBR)^17,18^. BBD is characterized by a dark polymicrobial mat that progresses across the host coral colony, killing coral tissue and exposing white skeleton^21^. The pathogenicity of BBD derives from the anoxic and sulphide-rich microenvironment created by the synergistic effects of a consortium of cyanobacteria, sulfur cycle-related bacteria, and other heterotrophic microorganisms present in the disease mat^22,23^. BrB, in contrast, is a much simpler disease caused by only one or two species of ciliates directly feeding on the coral tissue^24,25^. The macroscopic sign of BrB in the field is a brown zone flanked by healthy tissue at the advancing front and exposed white skeleton at the trailing edge^17^. Both diseases are readily transmitted among local coral hosts, however, the role of corallivores, such as butterflyfish (*Chaetodon* spp.), marine snails (e.g., Drupella spp.) and crown-of-thorns starfish (*Acanthaster* spp.), in transmitting coral diseases remains equivocal. In a few studies^19,26–28^, corallivorous vectors, predominantly gastropods, have been confirmed to actively transmit pathogens to new hosts within coral populations. However, most other corallivores appear to contribute to disease transmission indirectly, promoting pathogenic infections by weakening the host and/or creating an entry point for pathogens^29^. For example, the crown-of-thorns starfish, *Acanthaster planci*, is known to produce large feeding scars that can be the origin of BrB infections^30,31^. Observations that corallivorous fishes feed selectively on infected coral tissues led to speculation that they transmit coral diseases^32,33^. In experimental tests, the presence of butterflyfishes increased the transmission rate of BBD^34^, but this result may be attributable to nutrient enrichment rather than direct transmission; no study has explicitly demonstrated direct transmission of a coral pathogen by corallivorous fishes^28,34^.

Here we present a novel study evaluating the effects of predation by the gastropod *Drupella* (Muricidae) and coral-feeding *Chaetodon* butterflyfishes (Chaetodontidae) on the transmission rates of two common coral diseases on the GBR: BBD and BrB. Both aquarium and field-based experiments were used to provide a better understanding of the vector potential of *Chaetodon plebeius* and *Drupella* sp. Aquarium experiments were designed to explicitly test the hypothesis that corallivores directly transmit coral diseases by feeding successively on infected and uninfected corals. Building on results of a previous study, which demonstrated that the gastropod *Drupella* sp. is capable of transmitting BrB to corals^28^, the duration of the vector potential of *Drupella* was investigated by testing whether the snail could transmit BrB or BBD up to 24h after exposure to the disease. The potential of corallivorous reef fish to transmit BrB and BBD was also tested in the field: a) under natural rates of butterflyfish predation (uncaged treatment), and b) in the absence of predation (caged treatment). To synthesise new insights into whether and how vector-borne diseases circumvent density-dependent infection dynamics that prevent species extinctions, we review existing knowledge of coral disease vectors and their potential to amplify coral disease impacts on coral population dynamics. The review of the literature provides insight into whether the potential for disease transmission is stronger in vertebrate or invertebrate corallivores.

## Results

### Aquarium experiment: Potential of Drupella as a disease vector

#### Brown band disease experiment

**Figure 1:**
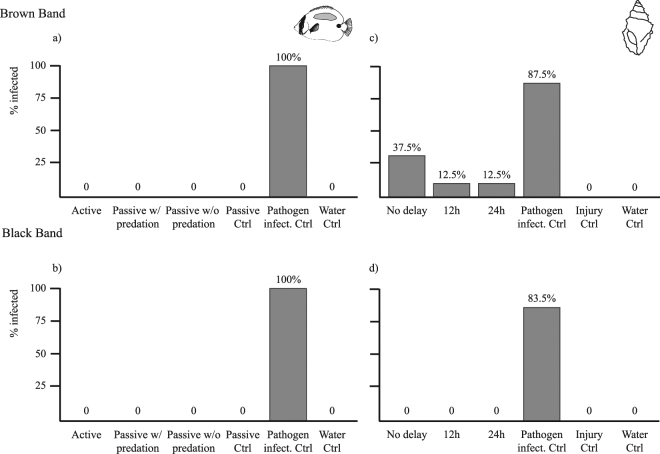
Percentage of nubbins infected in disease transmission experiments involving the butterflyfish *Chaetodon plebeius* (**a**,**b**) and the snail *Drupella* (**c**,**d**). The *C*. *plebeius* transmission experiment used a) brown band disease and b) black band disease, and comprised three treatments: active transmission, passive transmission with fish predation, and passive transmission without fish predation treatments, with three controls (passive transmission, pathogen infectivity, and seawater controls). The *Drupella* transmission experiment used c) brown band and d) black band disease, and comprised three treatments: a no delay introduction of snails to tanks, a 12 h delay, and a 24 h delay, with three controls (pathogen infectivity, injury and seawater controls).

*Drupella* snails transmitted BrB in 3 out of 8 replicates (~40%) in the “No delay” treatment, and in 1 out of 8 replicates (12.5%) in both the “12h delay” and “24h delay” treatments (Fig. 1). High rates of transmission (7/8) occurred in the pathogen infectivity control (“direct contact”), confirming that pathogens were active. No transmission was observed for either the seawater control or injury control nubbins. This signifies that new infections in the treatment tanks were a result of ciliates carried by *Drupella* snails and not caused by potential pathogens in the seawater system colonising feeding injuries. A generalized linear model and subsequent likelihood ratio test comparing “treatments” against “controls” (treatments pooled together and compared against pooled controls, except for the pathogen infectivity control, which was excluded) revealed that the presence of *Drupella* significantly increased infection rates of BrB in comparison to controls (Analysis of deviance table; Vector, DF_resid_ = 38, p = 0.02, Fig. 1). When *Drupella* treatments were compared against each other, infection rates did not differ among the ‘No delay’, ‘12h delay’ and ‘24h delay’ treatments (Analysis of deviance table; Treatment, DF_resid_ = 21, p = 0.38), suggesting that snails do not lose their vector potential over a 24h period. One nubbin in the seawater control treatment bleached and died, but no signs of ciliates or other pathogens were observed on the nubbin.

#### Black band disease experiment

BBD was never transmitted in any of the “No delay”, “12h delay” or “24h delay” *Drupella* treatments. All injury and seawater control nubbins remained healthy throughout the experiment, whereas 5 out of 6 pathogen infectivity controls became infected. One nubbin in the “12h delay” treatment became infected with BrB ciliates, even though the snail was exposed to BBD. The ciliates were unlikely to have come from the filtered seawater system since the injured and seawater controls remained healthy; instead, they may have been present on the snails since initial collection from the field (up to 3 days prior).

### Aquarium experiment: Potential of corallivorous fish as disease vectors

In aquaria, no transmission of BBD or BrB occurred between infected and closely positioned healthy nubbins of *A*. *muricata* in the presence of *C*. *plebeius*, which were observed feeding on the disease lesions and then the healthy nubbins. The only instances of disease transmission in these experiments occurred in pathogen infectivity controls, whereby 100% of seemingly healthy nubbins placed in direct contact with infected nubbins (5/5 nubbins for BrB and 7/7 for BBD) developed conspicuous signs of disease. This demonstrated that pathogens were active and infectious, and that new infections would be readily apparent within the 6-day duration of experiments. However, in all other treatments (3 fish treatments, passive transmission and water controls), no disease transmission was detected for either BBD or BrB. These data suggest that *C*. *plebeius* do little, if anything, to promote transmission of BRB or BBD.

### Field experiment: Potential of corallivorous fish as disease vectors

**Figure 2:**
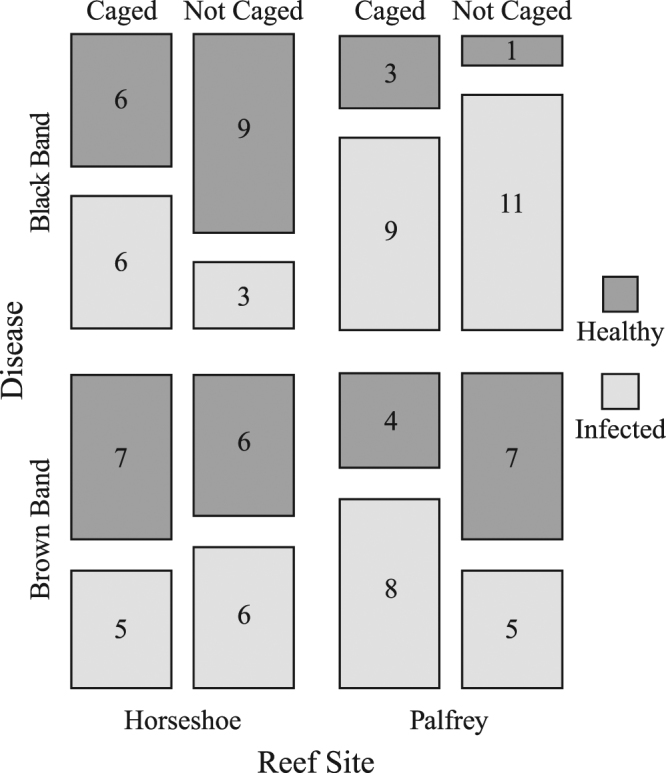
Table plot illustrating the proportion of healthy and infected (pathogen present) nubbins in relation to caging treatment, disease type and reef site. The field experiment ran for 7 days and monitored every second day. N = 96 nubbins overall.

In the field, 55% of experimental *A*. *muricata* branches (n = 96 branches) became infected with either brown band, black band or skeletal eroding band disease (another ciliate-related coral disease) during the 7-day observation period. Of the 51 branches that developed new infections, slightly more than half (28 branches) were caged and, therefore, protected from feeding by corallivorous fishes. Feeding observations on day 2 confirmed that several different species of corallivorous butterflyfishes (*Chaetodon aureofasciatus*, *C*. *baronessa*, *C*. *lunulatus* and *C*. *plebeius*) visited the experimental blocks, and fed on both the infected branches at the centre of the block and the uncaged branches on opposite corners of each block. Video footage confirmed that butterflyfishes were unable to access caged branches. Despite obvious differences in visitation and feeding by corallivorous butterflyfishes, caging had no effect on whether branches became infected (glmer Laplace approximation; Caging, z = −0.66, p = 0.51; Fig. 2). The number of infections was significantly higher at Palfrey Island than at Horseshoe Reef (glmer Laplace approximation; Site, z = 2.74, p = 0.006, Fig. 2).

### Literature Review

Results from 22 published studies were compiled to assess the capacity of corallivores to amplify the impact of diseases on coral populations by acting as vectors and facilitators of infections (Table 1). Only seven (out of 22) studies have experimentally demonstrated a corallivore to be an effective vector of coral disease. One unique disease, *Porites* trematodiasis, causes swollen nodules on corals^35–39^ as a consequence of infection by the trematode *Podocotyloides stenometra*, which requires multiple intermediate hosts (a mollusc, *Porites* corals, and the corallivorous fish, *Chaetodon multicinctus*) to complete its complex life cycle^38^. Of the remaining studies, only 6 have successfully identified a pathogen within the vector’s body, or have shown that vectors transmit disease in controlled experiments^19,26–28,40,41^. The majority of studies found correlations between disease onset and the presence of, or predation by, a corallivore but did not demonstrate a causal link^29–33,42–44^. Other controlled experiments found that corallivory did not increase coral disease transmission^28,34,41,45^. The majority of the studies reviewed were conducted in the Caribbean region, where the most successful vector is the marine snail *Coralliophila abbreviata*, which has been shown to transmit three diseases^19,27,41^. The close relative, *Coralliophila violacea*, was also shown to cause tissue loss (resembling white syndrome) in *Porites* in Guam after feeding on infected and healthy colonies, although the vector potential of the snail remains equivocal since secondary infections remain likely due to the experimental design^46^. In the Indo-Pacific, *Drupella* snails, and potentially crown-of-thorns starfish, are the most likely candidates as coral disease vectors^28,30,31,42^. Except in the case of trematodiasis, all corallivores experimentally proven to transmit coral diseases are invertebrates: *Hermodice carunculata* (Polychaeta), *Cyphoma gibbosum* (Gastropoda), *Coralliophila abbreviata* (Gastropoda), *Drupella* sp. (Gastropoda).

**Table 1:** List of peer-review publication aimed at testing the effect of potential vectors on coral disease transmission; listed by main finding, disease type, vector organism, pathogen species, transmission mechanism and source.

Finding	Disease	Vector	Pathogen	Mechanism	Source
Vector transmitted parasite	Trematodiasis	*Chaetodon multicinctus*	Podocotyloides stenometra	*P*. *stenometra* has a complex life cycle involving a molluscan first intermediate host, *Porites* coral as the second intermediate host, and coral-feeding fish as the final host	Aeby^35–39^
Pathogen detected within the vector’s body	Vibrio shiloi Bleaching	*Hermodice carunculata*	*Vibrio shiloi*	Worms contained viable *V*. *shiloi* bacteria and transmitted bleaching to healthy *Oculina patagonica*	Sussman *et al*.^26^
	Aspergillosis	*Cyphoma gibbosum*	*Aspergillus syndowii*	*A*. *syndowii* was found to survive through the digestive track of the snail. Viable spores and hyphae in snail faeces.	Rypien & Baker^40^
	Acroporid Serratiosis	*Coralliophila abbreviata*	*Serratia marcescens*	Bacterial strains from *C*. *abbreviate* successfully infected *Acropora palamata* in aquaria	Sutherland *et al*.^19^
Vector transmitted disease in controlled experiments	Unknown Disease	*Coralliophila abbreviata*	Unknown	Snails feeding on infected colonies transmitted disease to healthy nubbins	Williams & Miller^27^
	White Band Disease	*Coralliophila abbreviata*	*Vibrio* and *Rickettsiales* bacteria	Snails collected from the field transmitted WBD to healthy nubbins in aquaria	Gignoux-Wolfsohn *et al*.^41^
	Brown Band Disease	*Drupella* sp.	*Philaster guamensis*	Snails collected on infected colonies in the field transmitted BrB to healthy nubbins in the laboratory	Nicolet *et al*.^28^
Correlation between disease onset and either presence of or predation by vector	Coral Diseases	*Drupella cornus*	Various	Correlation between abundance of snail and diseases prevalence	Antonius & Riegl^42^
	Unknown Disease	*Phestilla sp*	Unknown	One nudibranch was placed on 7 coral fragments and progressive coral tissue mortality followed predation	Dalton & Godwin^43^
	Unknown Disease	*Hermodice carunculata*	Unknown	*H*. *carunculata* commonly observed feeding on disease margin	Miller & Williams^44^
	Coral Diseases	Chaetodontids	Various	Correlation between chaetodontids density and coral disease prevalence	Raymundo *et al*.^29^
	Black Band, Brown Band Disease	Chaetodontids	*P*. *guamensis*, bacterial consortium	*Chaetodon aureofasciatus*, *C*. *baronessa*, *C*. *lunulatus*, *C*. *plebeius* and *C*. *trifascialis* selectively targeted disease lesions over adjacent healthy coral tissues.	Cole *et al*.^32^, Chong-Seng *et al*.^33^
	Brown Band Disease	*Acanthaster planci*	*Philaster guamensis*	Feeding scars of crown-of-thrones starfish became the origin of BrB infections	Nugues & Bak^30^, Katz *et al*.^31^
Corallivore not found to transmit disease in controlled experiments	Black Band Disease	*Chaetodon capistratus*	Bacterial consortium	Feeding behaviour of the fish did not increase *Phormidium corallyticum* transmission	Aeby & Santavy^34^
	White Band Disease	*Coralliophila caribaea*	*Vibrio* and *Rickettsiales*	*C*. *caribaea* feeding behaviour did not transmit WBD in aquarium-based infection experiment	Gignoux-Wolfsohn *et al*.^41^
	White Syndrome	*Cyamo melanodactylus*	Unknown	Transplanting crabs from infected colonies onto healthy corals does not result in disease transmission	Pollock *et al*.^45^
	Brown Band Disease	*Chaetodon aureofasciatus*	*Philaster guamensis*	The fish neither aided nor hindered the transmission of BrB from infected to uninfected corals	Nicolet *et al*.^28^

## Discussion

This study demonstrates that *Drupella* snails transmit the virulent coral disease, brown band disease (BrB), both immediately after feeding and for at least 24 h after feeding on diseased coral nubbins. From a 40% maximum infection rate in direct transmission treatments (“no delay treatment”), the vector potential of *Drupella* declined, although not significantly, following 12h and 24h delays between disease exposure and introduction to healthy nubbins. Survival of BrB ciliates within the snail for 24 hours, and potentially longer, would facilitate disease transmission, both within and between coral colonies *in situ*. Considering the rapid progression rates reported for BrB (over 4 cm day^−1^;^31^), the ease with which *Drupella* transmits the disease, and the sheer magnitude of snail numbers reported for *Drupella* outbreaks (single aggregations up to 3000 snails per m^2^;^47^), the disease is capable of causing substantial mortality in host populations. The potential of vector-transmitted pathogens to drive significant population declines and even extinctions of host species suggests that managing *Drupella* outbreaks will be crucial to controlling potential disease outbreaks in coral populations. In contrast, *Drupella* did not transmit BBD and butterflyfish did not transmit either disease in the laboratory. Consistent with the laboratory study, predation by butterflyfish in the field experiment had no effect on the incidence of new infections.

The inability of *Drupella* snails to transmit BBD is most likely due to the complexity of the BBD pathogenic community. Whereas BrB is caused by one to two species of ciliates^24,25,48^, black band disease is characterised by a complex microbial mat that evolves through time from a cyanobaterial patch to a fully-developed polymicrobial BBD mat^22,49^. Hence, *Drupella* might not have the potential to carry all required microbes, in the right proportions, to establish BBD in a new host. While BBD is prevalent on reefs around the world, experimental and observational studies conducted with a range of potential vectors (*Drupella* sp., *Chaetodon capistratus*) have never found BBD to be vector-transmitted (^27,28,34^, present study). The one study purported to show that *Chaetodon* butterflyfishes (specifically, *C*. *capistratus*) contribute to transmission of BBD^34^, reported effective transmission in the presence of fishes, whether or not fish had access to nubbins. Direct transmission of BBD via corallivory is thus unlikely, potentially due to the complexity of the microbial community causing BBD.

Our study suggests that corallivorous butterflyfishes play a limited role in the transmission of either BBD or BrB. This conclusion is supported by aquarium experiments, where none of the experimental corals in butterflyfish treatments became infected. Even under high predation pressure, associated with four fishes feeding directly on both diseased and healthy nubbins for 6 consecutive days, corallivorous fishes were never found to initiate BBD or BrB. Similarly, in field experiments, we found no difference in the proportion of new infections between caged versus uncaged coral nubbins. Together, these findings suggest that corallivorous butterflyfishes are not effective vectors of coral diseases, which is supported by previous studies that never found butterflyfish feeding behaviour to increase either BrB or BBD transmission rate^28,34^. However, *Chaetodon multicinctus* do play an indirect role in the dynamics of one parasite infection, *Porites* trematodiasis, by being an intermediate host in the life cycle of the trematode (see^35–39^). In other infectious diseases, however, factors, such as environmental conditions or inherent variability in susceptibility among corals, are likely to influence transmission rates far more than the presence or absence of corallivorous fishes.

Differences in the capacity of invertebrate corallivores versus polyp-feeding butterflyfishes to contribute to the transmission of coral diseases could be related to their specific feeding behaviour, as these fishes rarely remove enough tissue to expose coral skeletons^50^. Many studies have emphasised the role of deep tissue injury and exposed skeleton in the spread of diseases, particularly BrB^28,30,31^, BBD^34^ and skeletal eroding band (SEB:^51,52^). In aquarium experiments testing for coral disease transmission, only corals with injuries exposing underlying skeleton became infected, regardless of experimental setting or disease type (BrB:^28,31^; SEB:^51^; BBD:^34^). We conclude that corallivores inflicting deep feeding scars, such as those caused by many invertebrates, are better candidates than butterflyfishes as vectors of coral diseases. Additional studies quantifying the depth of feeding scars from different invertebrate and vertebrate corallivores and verifying disease transmission are needed to test this hypothesis.

An extensive review of the literature on vectors of coral diseases highlights the paucity of confirmed reports. Only three studies have detected coral disease pathogens within the bodies of vectors^19,26,40^, and three additional publications have shown disease transmission to be possible through vectors in controlled experiments (^27,28,41^; Table 1). To date, only four vectors have been confirmed to transmit a total of six coral diseases^19,26–28,40,41^. Of the four confirmed coral disease vectors, the fireworm *Hermodice carunculata* acts as a winter reservoir and a summer vector of *Vibrio shiloi*, a bacterium causing bleaching^26^. *H*. *carunculata* was also observed feeding on disease lesion during an outbreak of a ‘white disease’ in the Caribbean^44^. The remaining three vectors are corallivorous gastropods (Table 1). The Caribbean snail *Coralliophila abbreviata* (accepted species name now *C*. *galea*) has successfully transmitted various bacteria responsible for acroporid serratiosis (*Serratia marcescens*), white band disease (*Vibrio* and *Rickettsiales* bacteria) and an unknown type of white syndrome^19,27,41^. Interestingly, a close relative, *Coralliophila caribaea*, was unable to transmit white band disease in the same laboratory conditions^41^. Feeding scars of a snail from the same genus, *Coralliophila violacea*, were also found to be the origin of *Porites* white syndrome, this time in the Indo-Pacific^46^. Another mollusc, *Cyphoma gibbosum*, is a successful vector of the fungus *Aspergillus syndowii* that affects gorgonian corals^40^. Finally, *Drupella* spp. transmitted BrB in a precursor to this study^28^, as well as in the current study, and is the only confirmed vector in the Indo-Pacific. Although only 4 invertebrate vectors have been confirmed, a comprehensive review identified 314 invertebrate species that feed directly on coral tissue, including 4 *Drupella* species, 10 *Coralliophila* species and 12 echinoderm species (starfish and sea urchins)^53^. Many of these species leave deep feeding scars and, considering the limited research on coral disease vectors and the extensive number of corallivorous invertebrates, the importance of vectors in coral disease transmission is likely underestimated.

Frequency-dependant diseases in terrestrial ecosystems have had devastating consequences for their host populations, driving species to extinction (e.g.^4,54^). Most coral diseases affect multiple coral species^16,17^, and vectors can maintain pathogen loads independently of host populations (e.g.^26^). Consequently, coral diseases have the potential to inflict significant losses on coral populations because pathogens are not constrained to decline as host density declines. Managing diseases in the natural environment requires knowledge of disease transmission mechanisms, aetiology and pathogenesis, but such knowledge is currently limited for coral diseases^55^. Even when the pathogen has been identified, diseases affecting corals are challenging to manage or treat directly due to the complexity of the holobiont and the nature of the marine environment. The results of this study show that invertebrate vectors that create relatively deep feeding scars are the most likely vectors of coral diseases, whereas disease transmission by corallivorous butterflyfishes would occur only rarely on coral reefs. Effective control of invertebrate corallivores (e.g.^56^) that are known to either cause (*Drupella* sp.) or facilitate (crown-of-thorns starfish) disease transmission would help to minimise the spread and prevalence of coral diseases. In the face of climate change and increasing collapse of coral reef ecosystems throughout the world, coral diseases are likely to play a crucial role in the dynamics of coral populations. Focus should be directed to understanding coral disease transmission mechanisms, particularly disease vectors, in order to moderate disease impacts on coral populations.

## Materials and Methods

### Ethics statement

All animal procedures followed strict guidelines set by James Cook University ethics committee and the Great Barrier Reef Marine Park authority. The project was approved by James Cook University ethics committee (ethics approval A1345, A1717, A2015) and was performed under James Cook University fisheries permit (103256) and Great Barrier Reef Marine Parks permit (G09/29157.1, G11/32003.1, G13/35909.1).

### Study location and study species

All experiments took place on Lizard Island (14˚40’08’’S 145˚27’34’’E), a mid-shelf island in the northern Great Barrier Reef, Australia. At Lizard Island, populations of the staghorn coral *Acropora muricata* had higher disease prevalence than any other coral species at the time of the experiments, especially of BBD and BrB (29, Nicolet *et al*. *in press MEPS*). This species was thus selected as the experimental coral for its susceptibility to disease and its local abundance.

### Aquarium set-up and maintenance of experimental animals

Experimental studies were conducted in flow-through aquaria at Lizard Island Research Station in March-June 2013 (BBD experiments) and January-March 2014 (BrB experiments). Different aquaria were used for disease transmission experiments using the corallivorous butterflyfish *Chaetodon plebeius* (120 × 40 × 50 cm aquaria) and the gastropod *Drupella* sp. (30 × 30 × 50 cm aquaria) due to the different requirements of these animals. All aquaria were supplied with flow-through seawater filtered to 0.5 μm and UV sterilized. *C*. *plebius*, *Drupella* sp., and nubbins of the coral *Acropora muricata* used in these experiments were all collected from within the Lizard Island lagoon. *C*. *plebeius* was used as it interacted most frequently with BBD and BrB lesions in video recordings from a previous study on the same reefs (Nicolet *et al*. *in press MEPS*). Adult and sub-adult *C*. *plebeius* (5 to 8 cm total length) were collected using a 5 × 1.5 m barrier net and hand nets. Healthy nubbins of *A*. *muricata* (between 15 and 20 cm length) were collected from various reefs within the lagoon, from colonies larger than 1 m in diameter, and the absence of BBD lesions or BrB ciliates confirmed under a dissecting microscope (Olympus SZX7, 50x magnification). Fish and coral nubbins were allowed to acclimate to aquarium conditions for 48 h prior to the experiment. *Drupella* snails were collected from rubble and *Acropora* thickets by hand using laboratory gloves, avoiding snails on or near disease lesions. All snails were placed in a holding tank (120 × 40 × 50 cm aquarium) containing diseased corals (either BBD or BrB) for a 3-day exposure period. After respective acclimation periods for fish and snails, heavily diseased (disease band wider than 0.5 cm) nubbins of *A*. *muricata* were collected from the reef and placed in experimental tanks as described below. Butterflyfish are limited in their energetic intake from any one coral (especially fragments), therefore, they require multiple fragments to ensure adequate access to prey. For this reason, *C*. *plebeius* were provided with healthy coral branches (renewed every 3 days) in addition to the diseased experimental nubbins. Corals were fed every day at dusk with brine shrimp (*Artemia salina* nauplii) hatched in 0.5 μm filtered and UV sterilized seawater. All healthy nubbins, diseased nubbins, fishes and snails from various reefs of origin were mixed in experimental aquaria to minimise any potential effect of parent colony or previous exposure of the animals to BBD or BrB.

### Aquarium experiment: Vector potential of the gastropod Drupella

After the 3-day period of exposure to either BrB or BBD, during which snails were observed to feed on diseased tissues, *Drupella* were placed in a holding tank for varying periods of time to determine how long pathogens might be retained and remain viable on the snail. Three experimental treatments were established to test the potential of *Drupella* to act as a vector for BrB and BBD (Supplementary material Fig. S1): (a) “No delay”, where 3 snails were placed in the holding tank for 5 seconds, then directly placed in an experimental tank at the base of a healthy nubbin; (b) “12 h delay”, where 3 snails were placed in contact with a healthy nubbin after spending 12 h in the holding tank; and (c) “24 h delay”, where 3 snails were placed in contact with a healthy nubbin after 24 h in the holding tank. Due to the discontinuous nature of feeding activity of the snails, immediate feeding could not be guaranteed; therefore, the “12 h” and “24 h” delayed treatments represent the minimum timespan between pathogen exposure and first feeding on coral hosts. Three controls for these experimental treatments comprised healthy and diseased nubbins in the absence of *Drupella* (Supplementary material Fig. S1), as follows: (d) an injury control, comprising a healthy nubbin mechanically injured with a sterilised scalpel blade, resulting in a 100 × 50 mm area where tissue was removed but the skeleton only minimally damaged to simulate a *Drupella* feeding scar without exposure to pathogen; (e) a pathogen infectivity control, comprising a diseased nubbin cable-tied to a healthy nubbin; and (f) a water control, comprising a single healthy nubbin in a tank to test for pathogen contamination in the aquarium system. Each trial comprised 6 tanks (1 tank per experimental or control treatment), and was replicated 8 times for the BrB experiment, and 6 times for the BBD experiment due to time and space constraints.

All *Drupella* snails were removed 48 h after the ‘24 h delayed transmission’ treatment was initiated, which was enough time to observe the presence or absence of snail feeding scars. All nubbins (both experimental and control) were monitored for another 3 days to allow any macroscopic signs of diseases to emerge. Each trial comprised of 9 snails and 7 nubbins (5 healthy, 1 injured and 1 infected; see Supplementary material Fig. S1). In total, experiments ran for 48 days for BrB (6 days per trial × 8 replicate trials) and 36 days for BBD (6 days per trial × 6 replicate trials). Initial statistical analyses showed that the “time” of these consecutive replicate trials had no effect on transmission, and ‘Time’ was therefore removed from the final statistical models. Between each replicate trial, all diseased, healthy and injured nubbins, and snails were replaced by new specimens collected from the field and acclimatised or exposed accordingly. A total of 72 *Drupella* were used for the BrB experiment (9 *Drupella* per trial × 8 trials between Jan—Mar 2014), and 54 *Drupella* were used for the BBD experiment (9 *Drupella* per trial × 6 trials between Mar–Jun 2013). Early.

### Aquarium experiment: Vector potential of the butterflyfish Chaetodon plebeius

To test whether the corallivorous butterflyfish (*C*. *plebeius*) is capable of transmitting BBD and/or BrB, and explore mechanisms by which potential transmission occurs, multiple fishes were placed in aquaria with and without access to diseased and healthy coral nubbins. Three experimental treatments distinguished between active versus passive transmission mechanisms (Supplementary material Fig. S2): (a) both healthy and diseased nubbins fully accessible to *C*. *plebeius* (4 fish per tank), testing for direct vectored transmission through successive feeding on diseased and then healthy nubbins (active transmission); (b) diseased nubbins accessible to *C*. *plebeius* (4 fish per tank) but healthy nubbins protected from predation by a semi-permeable tank divider, testing for passive transmission of pathogens due to dislodgement during feeding on diseased tissues and/or enhanced nutrients (passive transmission with feeding); and (c) neither diseased nor healthy nubbins accessible to *C*. *plebeius*, which were separated from the nubbins by a semi-permeable tank divider, testing whether the mere presence of the fishes increased transmission, possibly due to increased carbon and nitrogen levels (passive transmission without feeding). Controls for these treatments comprised diseased and healthy nubbins in the absence of fish (Supplementary material Fig. S2), as follows: (a) a healthy and an infected nubbin in a tank without direct contact (passive transmission control), (b) a diseased nubbin cable-tied to a healthy nubbin to test if BBD and BrB pathogens can infect corals in the aquarium setting (pathogen infectivity control), and (c) a single healthy nubbin in a tank to test for pathogen contamination in the aquarium system (water control).

Due to space limitations in the aquarium system, only one set of 3 experimental and 3 control treatments (hereafter referred to as a trial) could be conducted at any one time. Each trial ran for 6 days to allow enough time to detect the appearance of disease on healthy coral nubbins. Trials were replicated through time, i.e., 7 replicate trials for the BBD experiment (total of 8 aquaria per trial: 2 aquaria for each of the active and passive transmission with feeding treatments, 1 aquarium for the passive transmission without feeding treatment, and 1 aquarium for each of the passive transmission, pathogen infectivity, and water controls); and 5 times for the BrB experiment (again 8 aquaria per trial). The uneven design of the experiment (n = 2 for treatments with fish *versus* n = 1 for the treatment without fish during each replicate trial) and the time factor for the consecutive trials were accounted for in the statistical analyses. New fishes freshly caught from the reef replaced “used” fishes (after a 48h acclimation period) whenever possible. A total of 40 *C*. *plebeius* were used to run the 7 replicates of the BBD trial in Mar–Jun 2013, and another 30 *C*. *plebeius* for the 5 replicates of the BrB trial between Jan–Mar 2014.

### Field experiments: Vector potential of *in situ* assemblages of corallivorous fish

Field experiments testing the potential of *in situ* assemblages of large corallivores to transmit BBD and BrB were conducted in February 2009 at two Lizard Island sites: Horseshoe Reef on the western (leeward side) of the island, and a sheltered lagoon site between Palfrey and South Islands (Supplementary material Fig. S3). Nubbins of *Acropora muricata* (n = 96), approximately 10 cm long, were collected from healthy colonies in reefs on the north-west side of the island. One nubbin was attached to each corner of 24 concrete breezeblocks (39 cm × 18 cm) that had been conditioned by leaving them immersed in seawater for several weeks. Modelling clay was used to mount nubbins in plastic bottle tops attached to the concrete blocks with epoxy cement. Thus, each block contained four healthy unharmed experimental nubbins, one on each corner, for a total of 24 blocks and 96 experimental nubbins. An additional stressor treatment was originally added by bleaching half of the experimental nubbins using fresh water, however, bleaching treatment had no effect on disease transmission and thus, methods and results are not presented or discussed. To test if predation by large corallivorous fish enhances transmission of BrB or BBD to nearby nubbins, half of the healthy nubbins (2 on each block) were individually caged using plastic mesh with 1 × 1 cm openings (Supplementary material Fig. S3).

Blocks were deployed at the two reef sites and set 1–2 m apart at depths of 3–4 m. Half of the blocks were placed among the reef matrix at Horseshoe Reef (i.e., 12 blocks) and the other half within the sheltered lagoon between Palfrey and South Islands (i.e., 12 blocks). Both sites are similarly sheltered from the prevailing Southeast trade winds, and both had relatively high densities of corallivorous fishes known to target diseased corals^28^. Once blocks were positioned on the reef, an infected branch of *A*. *muricata* was mounted in the centre of each block (6 BrB-infected and 6 BBD-infected nubbins at each site), 20 cm away from uninfected branches. In summary, each experimental block held 5 nubbins (1 diseased nubbin, and 4 healthy nubbins, two of which were caged and 2 uncaged; Supplementary material Fig. S3). Blocks were surveyed every 2 days for 7 days to record the incidence of new infections. On day 2, video recordings (30 minutes per block) were also made to confirm that the cages effectively prevented corallivorous fishes from feeding on caged coral branches. At the end of the experiment, nubbins were brought back to the research station and observed with a dissecting microscope (Olympus SZX7, 50x magnification) for signs of infection.

### Statistical analysis

Disease transmission data from the *Drupella* experiment were analysed using generalized linear models, where “infection status” (binomial: infected or healthy) was the response variable, and the factor in the model was either “treatment” (5 levels) or “vector” (two levels: *Drupella* vs controls). The “treatment” factor levels were the three experimental treatments (no delay, 12 h and 24 h) and the two control levels (injury and water controls). The transmissibility control was excluded from the analysis because it was not directly related to corallivore vector potential, and was included only to ensure disease transmission was possible in tanks. For the model testing the “vector” factor, all transmission treatments were pooled together (*Drupella* level), and injury and seawater system controls were combined (control level). A likelihood ratio test was run on each model to compute p values (see supplementary material). No transmission was recorded in the chaetodontid experiments, either for BBD or BrB, and thus, the dataset was not formally analysed.

Data from the field experiments were analysed to test whether site of block deployment (Horseshoe vs. Palfrey), disease type (BrB versus BBD) and caging treatment (caged versus uncaged) influenced the incidence of infection using a generalised linear mixed model (Laplace approximation). The variable “status” referred to infection status and was treated as a binomial response variable in the analysis (infected or healthy, where infected indicates ciliate presence on nubbins). The random factor “block” was added to control for variation among replicates.

All statistical analyses were performed with R (version 3.0.2, R Development Core Team 2013). The generalized linear model used to analyse the aquarium experimental data was included in the ‘stats’ package, while the package ‘lme4’ was used to run the generalized linear mixed model to allow for random effects.

### Literature Review

To review the literature on coral disease vectors to date, the search term “coral disease vector” was used to collect all publications recorded in the ISI Web of Science database from 1965 to August 2016. Next, the literature was explored using references cited in relevant publications and broader search engines (e.g. Google scholar) to ensure the relevant publications were identified. Studies from this set of papers (n = 53 publications) were screened and only included if they focussed on biological vectors of coral diseases; studies on algal reservoirs of pathogens and non-biological vectors (e.g., ballast waters, dust) were not included. A total of 22 studies met the criteria for inclusion in this review.

### Data availability statement

All data generated or analysed during this study are included in this published article (and its Supplementary Information files)^13^.

## Electronic supplementary material

Supplementary material

Field Dataset

Drupella Dataset
