# Supplementary material for: Predation scars may influence host susceptibility to pathogens: evaluating the role of corallivores as vectors of coral disease
Source: Sci Rep. 2018 Mar 27;8:5258. doi: 10.1038/s41598-018-23361-y (PMC5869713; doi:10.1038/s41598-018-23361-y)
Supplement: Supplementary file 1 — Supplementary material [file 41598_2018_23361_MOESM1_ESM.pdf]

Supplementary Material

Manuscript Title: Predation scars may influence host susceptibility to pathogens: evaluating the role of corallivores as vectors of coral disease

K. J. Nicolet[1,2,3]*, K. M. Chong-Seng[2], M. S. Pratchett[2], B. L. Willis[1,2], M. O. Hoogenboom[1,2]

[1] College of Science and Engineering, James Cook University, Townsville, QLD 4811, Australia

[2] ARC Centre of Excellence for Coral Reef Studies, James Cook University, Townsville, QLD 4811, Australia

[3] AIMS@JCU, Townsville 4811, Australia

*Corresponding author

Katia Jane Nicolet

James Cook Drive 1, Townsville, QLD 4811, Australia

Email: katia.nicolet@my.jcu.edu.au

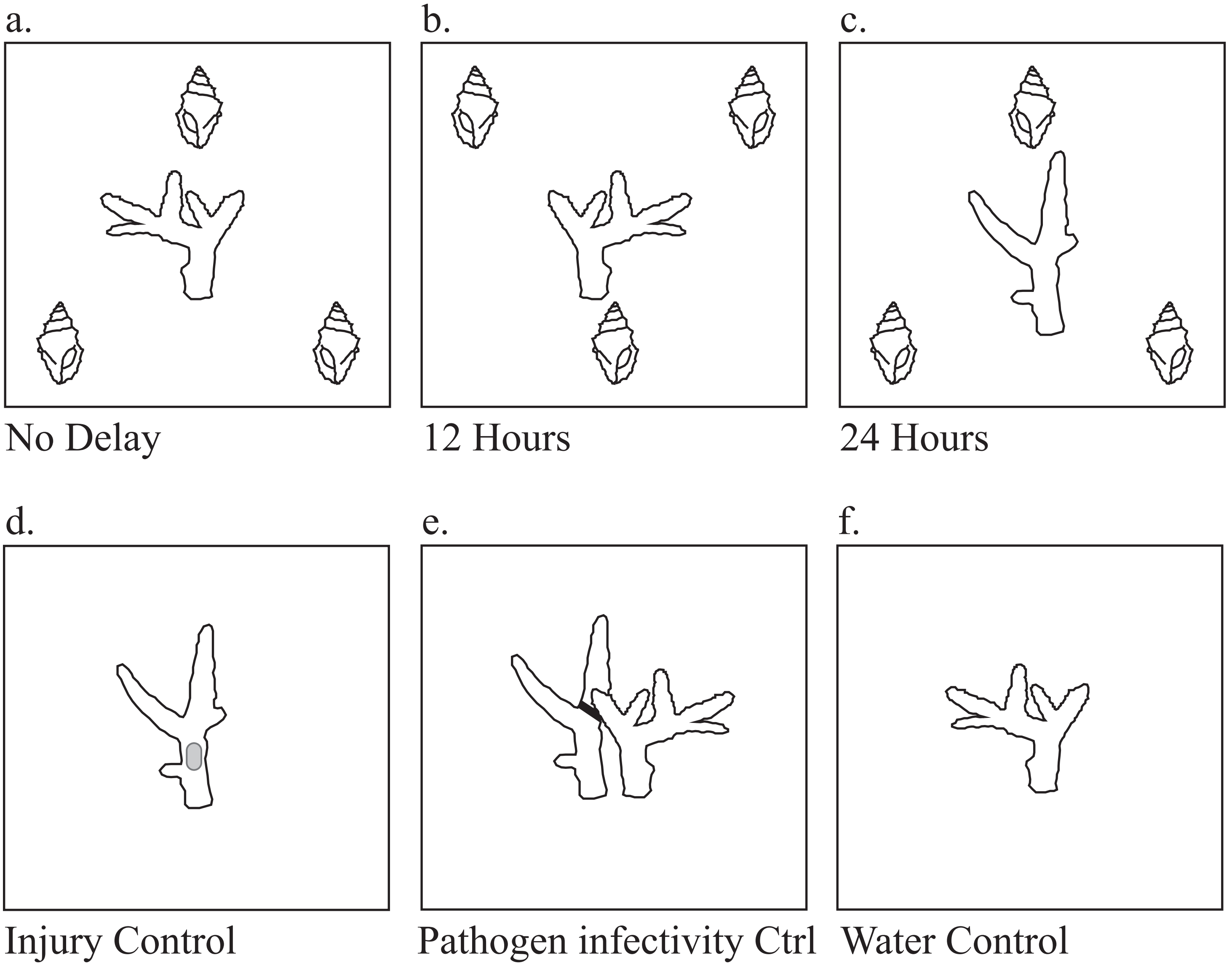

Supplementary Material Figure S1: Experimental design to assess the vector potential of *Drupella* snails for both BrB and BBD in aquaria. Prior to the experiment, the snails were fed nubbins heavily infected with either BrB or BBD for 3 days. Then, three individual snails were allocated to one of three treatments (a) no delay treatment, where snails were rinsed for 5 sec in filtered seawater before being introduced to the experimental tank; (b) 12h delayed treatment, where snails were kept in a holding tank for 12h before being placed in the treatment tank; (c) 24h delayed treatment, where snails were held in a similar fashion for 24h. Three controls were also added: (d) an injury control, comprising a healthy nubbin injured with a scalpel blade; (e) a pathogen infectivity control, comprising a diseased nubbin cabled-tied to a healthy nubbin; and (f) a water control, with a single healthy nubbin.

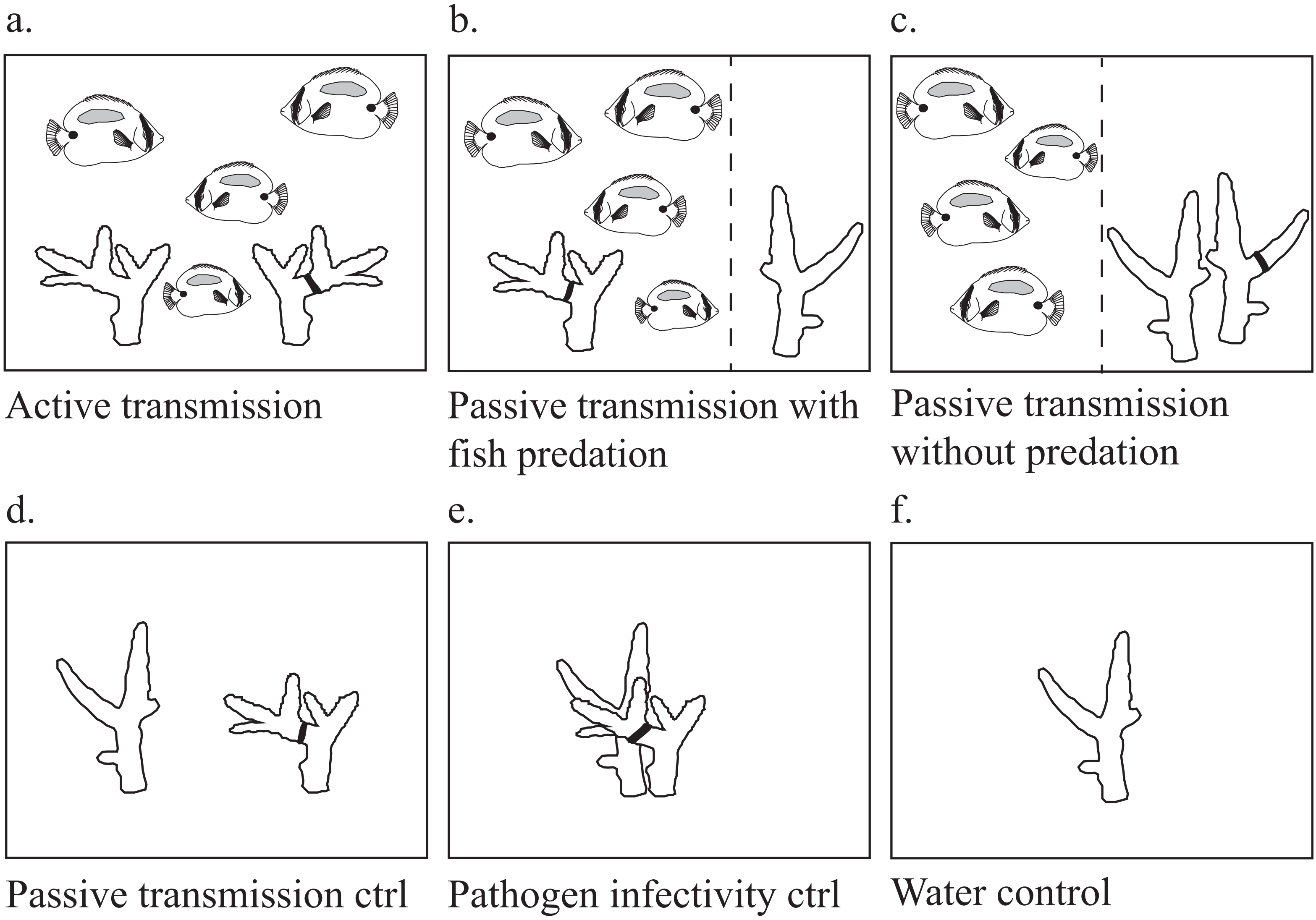

Supplementary Material Figure S2: Experimental design assessing the effect of predation by the butterflyfish *Chaetodon plebeius* on BBD and BrB transmission rates in an aquarium setting. Each replicate trial contained three treatments (a) active transmission, (b) passive transmission with fish predation, and (c) passive transmission without fish predation. The experiment also included three controls: (d) passive transmission without fish, (e) pathogen infectivity control, and (f) seawater system control.

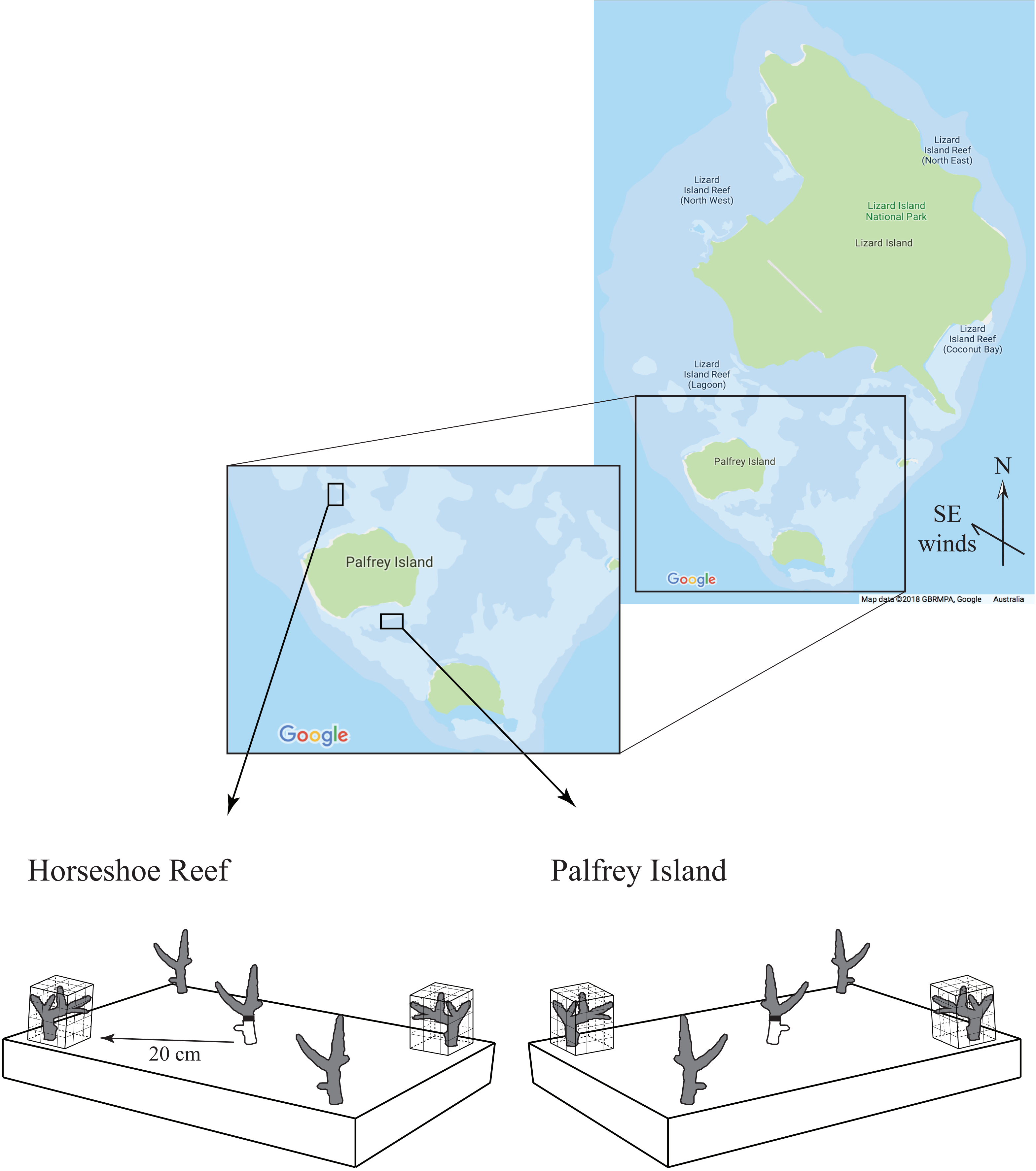

Supplementary Material Figure S3: Study sites and experimental design for the field experiment: Lizard Island, with arrows illustrating north and the prevailing wind direction, the two experimental sites, Horseshoe reef and South Palfrey Island, and experimental nubbins, either caged or uncaged, and location of the diseased nubbin in the middle of the block. Figure drawn by KJ Nicolet using Adobe Illustrator CC 2017 21.1.0 (©1987-2017), map imported from Google Maps (©2018).

R script Supplementary material

Katia Nicolet

20 December 2015

Field Experiment

Looking at the effect of predation on black band and brown band disease transmission rate.

In this dataset: "Status" is the response variable, the health status of the nubbins at the end of the experiment, either healthy of infected. The different factors in the dataset: "nubbin" refers to individual replicate branch; "reef" is the reef site, either Horseshoe or Palfrey; "disease" is the disease type of the infected nubbin in the centre of the block, either black band or brown band; "condition" is the condition treatment of the experimental nubbin before the onset of the experiment, either healthy or bleached in fresh water; "caging" refers to whether the nubbin was protected from predation by a cage "infection" is a detailed version of the response variable "Status". It lists the type of infections observed on the experimental nubbins at the end of the experiment. However, it was found to have no effect in preliminary statistical analysis. It was thus left out of the present analysis.

Data

```
infected<-read.csv("/Users/Katia/Desktop/Disease spread paper/Stats/Field/Excel and csv
files/field_infected.csv" summary(infected)
```

```
##      nubbin              reef            disease         block       condition
##  Min.   : 1.00   Horseshoe:48   black band:48   b1     : 8   bleached:48
##  1st Qu.:24.75   Palfrey  :48   brown band:48   b2     : 8   healthy :48
##  Median :48.50                                  b3     : 8
##  Mean   :48.50                                  b4     : 8
##  3rd Qu.:72.25                                  b5     : 8
##  Max.   :96.00                                  b6     : 8
##                                                 (Other):48
##      caging   infection      Status
##  caged  :47   Both: 7   healthy :42
##  uncaged:47   BrB : 9   infected:51
##  NA's   : 2   No  :42   NA's    : 3
##               SEB :32
##               NA's: 6
##
##
```

glmer Laplace approximation model with all factors included

```
library(lme4)
```

```
## Loading required package: Matrix
## Loading required package: Rcpp
```

```r
FIELD.glmerL1<-glmer(Status~caging+condition+reef+(1|block),data=infected,family='binomial')

summary(FIELD.glmerL1)
```

```
## Generalized linear mixed model fit by maximum likelihood (Laplace
##   Approximation) [glmerMod]
##  Family: binomial  ( logit )
## Formula: Status ~ caging + condition + reef + (1 | block)
##    Data: infected
##
##      AIC      BIC   logLik deviance df.resid
##    124.2    136.7    -57.1    114.2       86
##
## Scaled residuals:
##     Min      1Q  Median      3Q     Max
## -2.2834 -0.8505  0.4506  0.8126  1.7110
##
## Random effects:
##  Groups Name        Variance Std.Dev.
##  block  (Intercept) 0.4502   0.6709
## Number of obs: 91, groups:  block, 12
##
## Fixed effects:
##                  Estimate Std. Error z value Pr(>|z|)
## (Intercept)        0.1767     0.5229   0.338   0.7355
## cagingcaged       -0.3084     0.4660  -0.662   0.5081
## conditionhealthy  -0.8303     0.6081  -1.365   0.1721
## reefPalfrey        1.2524     0.4930   2.540   0.0111 *
## ---
## Signif. codes:  0 '***' 0.001 '**' 0.01 '*' 0.05 '.' 0.1 ' ' 1
##
## Correlation of Fixed Effects:
##             (Intr) cgngnc cndtnh
## cagingcagd -0.440
## condtnhlthy -0.557  0.014
## reefPalfrey -0.356 -0.013 -0.079
```

Caging is not significant
Disease not significant in itself but has an interaction with reef
Reef and block are significant
The interaction betweeen caging and condition is not significant

glmer Laplace approximation with only status, condition and reef as factors and block as random factor.

```r
library(lme4)

FIELD.glmerL2<-glmer(Status~condition+reef+(1|block),data=infected,family='binomial')

summary(FIELD.glmerL2)
```

```
## Generalized linear mixed model fit by maximum likelihood (Laplace
##   Approximation) [glmerMod]
##  Family: binomial  ( logit )
## Formula: Status ~ condition + reef + (1 | block)
##    Data: infected
##
##      AIC      BIC   logLik deviance df.resid
##    123.4    133.5    -57.7    115.4       89
##
## Scaled residuals:
##     Min      1Q  Median      3Q     Max
## -2.2096 -0.7995  0.4526  0.8004  1.9256
##
## Random effects:
##  Groups Name        Variance Std.Dev.
##  block  (Intercept) 0.5827   0.7633
## Number of obs: 93, groups:  block, 12
##
## Fixed effects:
##                      Estimate Std. Error z value Pr(>|z|)
## (Intercept)        -0.0002163  0.4934447   0.000  0.99965
## conditionhealthy -0.8010346  0.6454380  -1.241  0.21458
## reefPalfrey        1.3348739  0.4878159   2.736  0.00621 **
## ---
## Signif. codes:  0 '***' 0.001 '**' 0.01 '*' 0.05 '.' 0.1 ' ' 1
##
## Correlation of Fixed Effects:
##             (Intr) cndtnh
## condtnhlthy -0.619
## reefPalfrey -0.380 -0.091
```

```
anova(FIELD.glmerL2,test="LRT")
```

```
## Analysis of Variance Table
##           Df  Sum Sq Mean Sq F value
## condition  1  0.984   0.984   0.984
## reef       1  8.241   8.241   8.241
```

Only reef site is significant. The bleaching has no effect.

Model testing

Overdispersion

```
overdisp_fun<-function(model)
  {vpars<-function(m)
    {nrow(m)*(nrow(m)+1)/2}
  model.df<-sum(sapply(VarCorr(model),vpars))+length(fixef(model))
  rdf<-nrow(model.frame(model))-model.df
  rp<-residuals(model,type="pearson")
  Pearson.chisq<-sum(rp^2)
```

```
  prat<-Pearson.chisq/rdf
  pval<-pchisq(Pearson.chisq,df=rdf,lower.tail=FALSE)
  c(chisq=Pearson.chisq,ratio=prat,rdf=rdf,p=pval)}

overdisp_fun(FIELD.glmerL2)
```

```
##       chisq      ratio        rdf           p
## 80.2652383  0.9018566 89.0000000  0.7346758
```

p values isn't significant so we don't have overdispersion in the data.

R^2 for glmerL2 model

```
totalss<-var(resid(FIELD.glmerL2,type='pearson')+predict(FIELD.glmerL2,type='link'))
1-var(residuals(FIELD.glmerL2,type='pearson'))/(totalss)
```

```
## [1] 0.5649683
```

By removing caging and disease, we reduced the R^2 a little.

Drupella Experiment

This dataset only includes the BrB data (since BBD was never transmitted by Drupella or Chaetodontids)

Generalized linear model testing the effect of treatments and controls on coral disease infection rate. "Status" refers to the health of the coral nubbin at the end of the trial (healthy or infected) and "Factor" has two levels, Drupella (Direct, 12h and 24h pooled together) and the controls (injury and water control combined). "Treatment" containes all 5 treatments and tests whether infection rate differs between them.

```
DRUP<-read.csv('/Users/Katia/Desktop/Disease spread paper/Stats/Drupella/Excel and csv
files/Drupella BrB.csv summary(DRUP)
```

```
##    Treatment Status      Factor
##   12h    :8   H:35   Control :16
##   24h    :8   I: 5   Drupella:24
##   Direct:8
##   Injury:8
##   Water :8
```

```
DRUP.glm<-glm(Status~Treatment+Factor,family=binomial, data=DRUP)
summary(DRUP.glm)
```

```
##
## Call:
## glm(formula = Status ~ Treatment + Factor, family = binomial,
##     data = DRUP)
##
## Deviance Residuals:
```

```
##       Min        1Q    Median        3Q       Max
## -0.96954  -0.51678  -0.00008  -0.00008   2.03933
##
## Coefficients: (1 not defined because of singularities)
##                  Estimate Std. Error z value Pr(>|z|)
## (Intercept)     -1.946e+00  1.069e+00  -1.820   0.0687 .
## Treatment24h    -1.908e-15  1.512e+00   0.000   1.0000
## TreatmentDirect  1.435e+00  1.295e+00   1.108   0.2677
## TreatmentInjury -1.762e+01  3.802e+03  -0.005   0.9963
## TreatmentWater  -1.762e+01  3.802e+03  -0.005   0.9963
## FactorDrupella          NA         NA      NA       NA
## ---
## Signif. codes:  0 '***' 0.001 '**' 0.01 '*' 0.05 '.' 0.1 ' ' 1
##
## (Dispersion parameter for binomial family taken to be 1)
##
##     Null deviance: 30.142  on 39  degrees of freedom
## Residual deviance: 22.642  on 35  degrees of freedom
## AIC: 32.642
##
## Number of Fisher Scoring iterations: 18
```

```
anova(DRUP.glm,test="LRT")
```

```
## Analysis of Deviance Table
##
## Model: binomial, link: logit
##
## Response: Status
##
## Terms added sequentially (first to last)
##
##
##           Df Deviance Resid. Df Resid. Dev Pr(>Chi)
## NULL                         39     30.142
## Treatment  4      7.5        35     22.642   0.1117
## Factor     0      0.0        35     22.642
```

Injection rate does not significantly vary between the 5 treatment groups. Meaning that the Direct treatment group is not significantly different from the 12h, 24h, injury or water control group.

To compare only the Drupella treatments against each other, we tested only a subset of the data:

```
SUB.DRUP<-subset(DRUP,Factor=="Drupella"&Treatment%in%c("Direct","12h","24h"))
DRUP.glm1<-glm(Status~Treatment,data=SUB.DRUP,family=binomial)

summary(DRUP.glm1)
```

```
##
## Call:
## glm(formula = Status ~ Treatment, family = binomial, data = SUB.DRUP)
##
## Deviance Residuals:
```

```
##      Min       1Q   Median       3Q      Max
## -0.9695  -0.5168  -0.5168  -0.5168   2.0393
##
## Coefficients:
##                   Estimate Std. Error z value Pr(>|z|)
## (Intercept)     -1.946e+00  1.069e+00  -1.820   0.0687 .
## Treatment24h     1.635e-15  1.512e+00   0.000   1.0000
## TreatmentDirect  1.435e+00  1.295e+00   1.108   0.2677
## ---
## Signif. codes:  0 '***' 0.001 '**' 0.01 '*' 0.05 '.' 0.1 ' ' 1
##
## (Dispersion parameter for binomial family taken to be 1)
##
##     Null deviance: 24.564  on 23  degrees of freedom
## Residual deviance: 22.642  on 21  degrees of freedom
## AIC: 28.642
##
## Number of Fisher Scoring iterations: 4
```

```r
anova(DRUP.glm1,test="LRT")
```

```
## Analysis of Deviance Table
##
## Model: binomial, link: logit
##
## Response: Status
##
## Terms added sequentially (first to last)
##
##
##           Df Deviance Resid. Df Resid. Dev Pr(>Chi)
## NULL                         23     24.564
## Treatment  2   1.9219        21     22.642   0.3825
```

Even when comparing only the Drupella treatments against each other, no significant difference in infection rate could be found. Meaning that the BrB infection rate in the direct treatment is not significantly different from the infection rate in the 12h or 24h treatment.

So infection rate after 12h and 24h is similar to infection rate in the direct treatment.

Now we want to know if the presence of Drupella significantly affects the nubbins infection rate. To test this, we pooled all the Drupella treatments together and tested them against the combined controls.

Model with just Factor: Drupella (Direct, 12h and 24h) against Control (Injury, Water)

```r
DRUP.glm2<-glm(Status~Factor,family=binomial, data=DRUP)
summary(DRUP.glm2)
```

```
##
## Call:
## glm(formula = Status ~ Factor, family = binomial, data = DRUP)
##
```

```
## Deviance Residuals:
##      Min        1Q    Median        3Q       Max
## -0.68354  -0.68354  -0.00008  -0.00008   1.77122
##
## Coefficients:
##                Estimate Std. Error z value Pr(>|z|)
## (Intercept)      -19.57    2688.50  -0.007    0.994
## FactorDrupella    18.23    2688.50   0.007    0.995
##
## (Dispersion parameter for binomial family taken to be 1)
##
##     Null deviance: 30.142  on 39  degrees of freedom
## Residual deviance: 24.564  on 38  degrees of freedom
## AIC: 28.564
##
## Number of Fisher Scoring iterations: 18
```

Check for Goodness of fit and Overdispersion

```
DRUP2.resid<-sum(resid(DRUP.glm2,type="pearson")^2)
1-pchisq(DRUP2.resid,DRUP.glm2$df.resid)
```

```
## [1] 0.9625835
```

```
1-pchisq(DRUP.glm2$deviance,DRUP.glm2$df.resid)
```

```
## [1] 0.9548658
```

```
DRUP2.resid/DRUP.glm2$df.resid
```

```
## [1] 0.6315789
```

```
DRUP.glm2$deviance/DRUP.glm2$df.resid
```

```
## [1] 0.6464085
```

```
#No lack of fit and no overdispersion

anova(DRUP.glm2,test="LRT")
```

```
## Analysis of Deviance Table
##
## Model: binomial, link: logit
##
## Response: Status
##
## Terms added sequentially (first to last)
##
##
##        Df Deviance Resid. Df Resid. Dev Pr(>Chi)
```

```
## NULL                      39     30.142
## Factor  1   5.5781        38     24.564  0.01819 *
## ---
## Signif. codes:  0 '***' 0.001 '**' 0.01 '*' 0.05 '.' 0.1 ' ' 1
```

Drupella has a significant effect on BrB infection rate. Meaning that the infection rate was significantly higher in the Drupella treatments than in the controls.
